# Ag/MXene as Saturable Absorber for Tm:Ho Co-Doped Q-Switched Fiber Laser

**DOI:** 10.3390/nano14110951

**Published:** 2024-05-29

**Authors:** Xiaoli Zhao, Jingxuan Sun, Yachen Wang, Xiaogang Wang, Bo Fu

**Affiliations:** 1 Key Laboratory of Precision Opto-Mechatronics Technology, School of Instrumentation and Optoelectronic Engineering, Beihang University, Beijing 100191, China; 2 Key Laboratory of Big Data-Based Precision Medicine Ministry of Industry and Information Technology, School of Engineering Medicine, Beihang University, Beijing 100191, China

**Keywords:** Ag/MXene composite, saturable absorber, Q-switched pulses, Tm:Ho co-doped fiber laser

## Abstract

Q-switched fiber lasers have become reliable light sources for generating high-energy pulses, which can be passively modulated by saturable absorbers with excellent nonlinear optical properties. The composite combining Ag and MXene exhibits a broadband nonlinear response and high modulation depth, making it a promising candidate for saturable absorbers in pulsed lasers. Herein, we demonstrate a Q-switched Tm:Ho co-doped fiber laser centered at 2 µm, where the Ag/MXene composite serves as a saturable absorber to generate pulses. The typical spectrum, pulse train, and radio frequency spectrum of Q-switched pulses were observed, in which the 60 dB signal-to-noise ratio was higher than that of 2 µm Q-switched fiber lasers based on other materials, demonstrating the stability of the output pulses. Additionally, the long-term stability of the laser was evaluated over 2 h, where the well-maintained central wavelength and output power also indicated the robustness of the Q-switched laser. Furthermore, the influence of the pump power on the parameters of Q-switched pulses was also investigated, which is conducive to control the output characteristics of lasers. Specifically, the pulse width of the Q-switched pulse decreased, while the repetition rate, output power, and single pulse energy all increased with the increase in pump power. These experimental results demonstrate the ability of Ag/MXene as a saturable absorber and show its potential for generating high-performance pulses in ultrafast lasers.

## 1. Introduction

Fiber lasers have attracted wide interest in various fields due to the advantages of a high beam quality, low cost, fast heat dissipation, and high stability [1]. Ultrafast pulses in fiber lasers are usually generated by Q-switching and mode locking, which have the advantages of a high peak power and ultrashort pulse width. Moreover, 2 μm pulsed lasers are in the eye-safe and water absorption waveband, which have been widely used in remote sensing, material processing, gas analysis, and medical treatment [2,3,4,5]. Q-switched lasers can satisfy the requirement of areas such as imaging and micro-machining owing to their high peak power, ultrashort pulse width, and pulse energy [6,7]. Q-switching can be generated by active or passive methods, where passive modulation has the advantages of a compact structure and easy adjustment. A saturable absorber (SA) with nonlinear saturable absorption characteristics stands out as a crucial component employed for the passive modulation of Q-switched pulse. Currently, 2-μm Q-switched fiber lasers have been achieved by many kinds of SAs, including artificial and real SAs. The primary differences between artificial and real SAs used in lasers lie in the composition and mechanism of saturable absorption effects of the SA devices. Artificial SAs are typically realized by a combination of optical components, where the saturable absorption is induced by the nonlinear effect or birefringent properties, such as nonlinear polarization rotation and nonlinear amplifying loop mirror. Artificial SAs used in lasers are easily affected by the surrounding environment and also have a complex structure. Therefore, other types of SAs still need to be studied, especially real SAs with saturable absorption properties. Typical real SAs include semiconductor saturable absorber mirror and nanomaterials, such as metal nanoparticles, carbon nanotubes, graphene, topological insulator, black phosphorus, and transition-metal carbides/nitrides/carbonitrides (MXene) [8,9,10,11,12]. The saturable absorption characteristics towards incident light of real SAs are from the materials themselves. Nanomaterials have the advantages of a broadband nonlinear absorption, ultrafast recovery time, and high environmental stability, which have been used as SAs in 2-μm laser systems to generate Q-switched pulses [11,12,13,14]. For example, in 2016, Yu et al. first demonstrated a Q-switched laser based on black phosphorus as the SA, where the 731 ns Q-switched pulse was obtained in 2 μm [15]. In 2019, Muhammad et al. obtained a Q-switched fiber laser at 1955 nm, which was based on a new titanium thin film as the SA [16]. In 2023, Al-Hiti et al. first reported a passively Q-switched thulium-doped fiber laser utilizing tungsten oxide as the SA, in which the output power and pulse energy were up to 8.2 mW and 127.6 nJ [17]. As a kind of two-dimensional material, MXene possesses outstanding nonlinear optical responses that are crucial for generating ultrafast pulses [18,19,20]. In addition, MXene has excellent properties in combination with other materials, which provides more possibilities for ultrafast applications [21,22,23,24]. Xie et al. designed and fabricated a composite of MXene nanosheets and gold nanorods, which enhanced the electromagnetic mechanism and chemical mechanism, thus improving surface-enhanced Raman scattering effect [21]. Ramlan et al. integrated a new Ag-doped indium selenide-based SA in fiber laser, and the experimental results validated and improved the optical properties of the Ag-doped indium selenide composite material [25]. Moreover, Ag possesses ultrafast recovery time and a large third-order nonlinear coefficient, and the study of ultrafast laser based on Ag as a saturable absorber at 2 μm has been reported [26,27]. For example, Lv et al. utilized Ag nanowires as saturable absorber to modulate lasers, where ultrafast pulses centered at 1967.4 nm were obtained for a Tm-doped fiber laser [27]. The combination of MXene and Ag is expected to exhibit superior optical properties and generate high-performance pulses in ultrafast lasers. Therefore, we explored the saturable absorption properties of the composite at 2 μm band and the performance of output pulses enabled by a Ag/MXene SA.

Herein, we demonstrate a Q-switched Tm:Ho co-doped fiber laser at 2 μm based on Ag/MXene as the SA. The nonlinear saturable absorption properties of Ag/MXene composite are characterized. Then, the typical results of Q-switched pulses based on the Ag/MXene SA are discussed in detail, such as the pulse train, repetition rate, output power, and pulse energy. The changes in these parameters versus the pump power are also observed. Moreover, the stability of the 2 μm laser is evaluated by observing its wavelength and output power over 2 h, and the results shows that the Q-switched fiber laser is robust in long-term operation. Furthermore, compared with the 2 μm results based on other materials, the enhancement of the Q-switched laser based on Ag/MXene composite is also demonstrated.

## 2. Experimental Methods

### 2.1. Properties of Ag/MXene Composite

The Ag/MXene composite was synthesized by a layer-by-layer spraying technology. The concentration of the original MXene and Ag solution was 2 mg/mL and 1 mg/mL, respectively. Then, the dispersion solution was sprayed by a sprayer at a distance of 10 cm and a pressure of 2 bar as evenly as possible. The Ag microparticles was firstly sprayed with an area density of 5 mg/cm^2^ in one waterborne polyurethane film, the dispersion solution of MXene was subsequently deposited on a Ag layer with an area density of 1 mg/cm^2^, and another waterborne polyurethane film was finally sprayed on the top [28].

**Figure 1:**
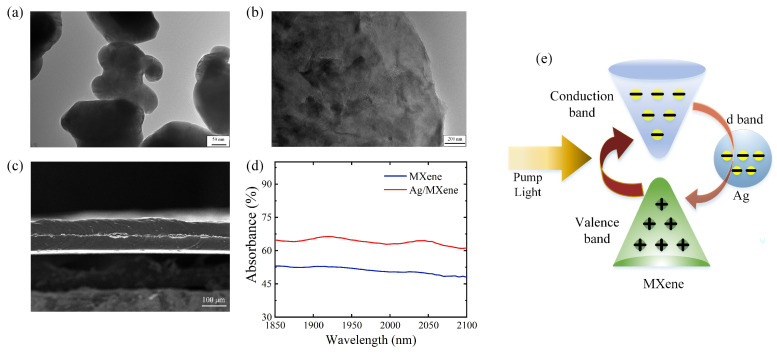
Characterizations of Ag/MXene composite. Transmission electron microscope images of (**a**) Ag and (**b**) MXene. (**c**) Scanning electron microscope image of the cross section of Ag/MXene composite. (**d**) Absorbance curves of MXene and Ag/MXene composites in the 2 μm band. (**e**) Physical mechanism of interaction between Ag and MXene.

The transmission electron microscope images of Ag nanoparticles and MXene nanosheets are shown in Figure 1a,b. The cross-section scanning electron microscope image of the Ag/MXene composite is shown in Figure 1c, which highlights that the thickness of the composite was around 125 μm and the core layer of Ag and MXene was around 9 μm. Theoretically, the increase in thickness of the core layer material increases the modulation depth ($\alpha_{s}$) and saturation intensity ($I_{sat}$) of the composite. However, the inherent losses ($\alpha_{ns}$) introduced by the preparation process or material itself also increase, while the higher saturation intensity also indicates the high threshold of pulse generation. Therefore, the thickness of the material is generally prepared on the order of tens of micrometers in experiments. In addition, the waterborne polyurethane film has a relatively high transmittance of light, and thus its effect on light transmission can be ignored. The absorbance curves of the composite and MXene to light were also measured, as shown in Figure 1d, which exhibits the uniform absorption of the MXene-based composite and its potential to modulate light in 2 μm laser. Specifically, the operation wavelength of the material is usually determined by its properties, which is mainly relevant to the bandgap of materials [29]. As a kind of metal material, Ag has a bandgap that can be considered as zero, and MXene possesses a narrow bandgap of <0.2 eV, suggesting a possible broad operation band of the composite material [30,31]. The MXene used in the experiment was Ti_3_C_2_(OH)_2_, where the work function of MXene is relatively low owing to the -OH functional group, further indicating its low surface electron affinity [32,33]. The work function of pure silver is about 4.3 eV, while the purity, contaminant, or crystal structure will affect the actual value [34,35,36]. As for the energy barrier, the heterointerface between MXene and another material has shown its ability to decrease the energy barrier, indicating the potential of a low-energy barrier of the composite material [37]. It can also be found that the energy barrier between composites is adjustable and affected by many factors such as doping and number of layers [38,39,40,41,42]. Moreover, considering that the saturable absorption property is related to the electronic transition of materials, the mechanism of interaction between Ag and MXene from the view of the electronic transition is illustrated in Figure 1e. The interaction between Ag and MXene affects the process of the excited electron transition [43]. In this regard, the electrons in MXene jump from the valence to conduction band under the excitation of a pump light, thereby realizing the absorption and transmittance to light. The combination with Ag prolongs the time for the excited electrons to return from the conduction band to the valence band, since the d band with a larger state density in Ag attracts electrons. Based on this process, the ability of the composite to modulate light is further strengthened, thereby enhancing the saturation absorption property. In addition, the third-order nonlinear polarization $\chi^{\left(3\right)}$ of the material may be affected by excited carriers generated at the leading edge of the laser pulse, which also contributes to the dependence of the saturation absorption response on the laser intensity [44]. Therefore, the composite of Ag and MXene is a potential SA candidate in the 2 μm band and is expected to exhibit outstanding saturable absorption under the interaction between Ag and MXene.

**Figure 2:**
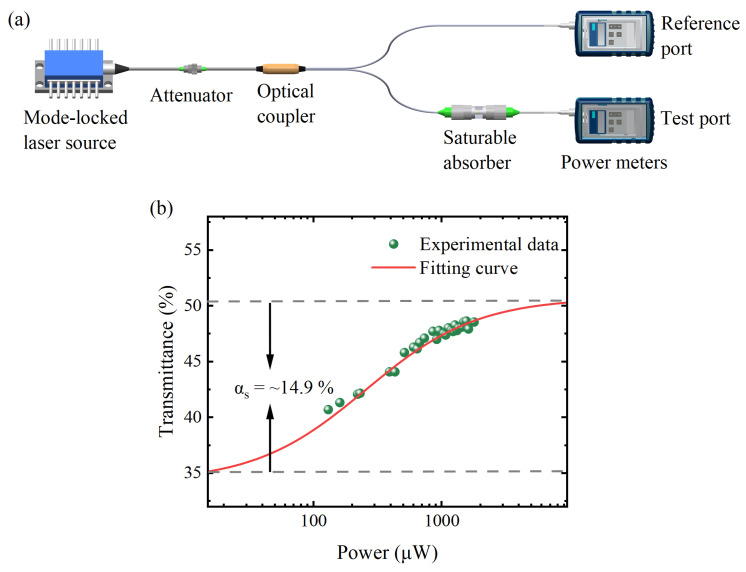
Nonlinear characterizations of Ag/MXene SA. (**a**) Setup of the balanced twin-detector measurement system. (**b**) Nonlinear transmittance results of Ag/MXene SA in the 2 μm band.

The sandwiched structure was applied to integrate the Ag/MXene composite as an SA device, and the composite was inserted between two fiber ferrules through an adapter. Then, the nonlinear saturable absorption property of the Ag/MXene SA was measured by a balanced twin-detector measurement system, as shown in Figure 2a. The mode-locked laser source was connected with a tunable optical attenuator, and then the light was divided into two beams by a 50/50 coupler. One reference beam was measured by a power meter directly, and the other test beam passed through the Ag/MXene composite and was then monitored by another power meter. The output powers of both arms changed along with the continuous adjustment of the attenuator. The ratio of the test port to the reference port power was the nonlinear transmittance. The experimental data were fitted by the following formula [45]

(1) $$T=1-\alpha_{s}/\left(1+I/I_{sat}\right)-\alpha_{ns}$$

where *T* is the nonlinear transmission, $\alpha_{s}$ and $\alpha_{ns}$ are the modulation depth and non-saturable component, and *I* and $I_{sat}$ represent the incident and saturable intensity. The experimental data and fitting curve of the nonlinear transmittance of Ag/MXene SA are shown in Figure 2b, where the modulation depth is defined as the maximum variation in light transmittance. It can be calculated from the fitting curve that the $I_{sat}$ and $\alpha_{s}$ of the Ag/MXene SA at 2 μm are 310 μW and 14.9%, respectively.

### 2.2. Laser Setup

**Figure 3:**
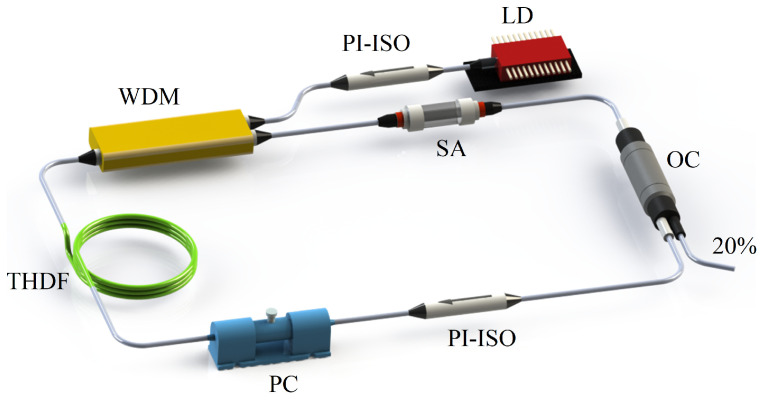
Setup of the 2 μm Q-switched fiber laser based on a Ag/MXene saturable absorber. LD: laser diode; PI-ISO: polarization-independent isolator; WDM: wavelength-division multiplexer; THDF: Tm: Ho co-doped fiber; PC: polarization controller; OC: optical coupler; SA: saturable absorber.

The 2 μm Q-switched fiber laser is schematically depicted in Figure 3. The gain medium was a 2 m Tm:Ho co-doped fiber, which was pumped by a 1550 nm continuous wave laser through a wavelength-division multiplexer. The unidirectional operation was ensured by using a polarization-independent isolator, while a polarization controller was employed to optimize the Q-switched operation and polarization state within the laser cavity. The Ag/MXene composite was integrated as an SA with a sandwiched structure. An optical coupler was utilized to extract 20% of the power to measure. The output was monitored by an optical spectrum analyzer (APE Wavescan USB, APE GmbH, Berlin, Germany), a frequency spectrum analyzer (Keysight N9020A, Santa Clara, CA, USA), an oscilloscope (Keysight DSOX1024G, Santa Clara, CA, USA), and pyroelectric optical power meters.

## 3. Results and Discussion

**Figure 4:**
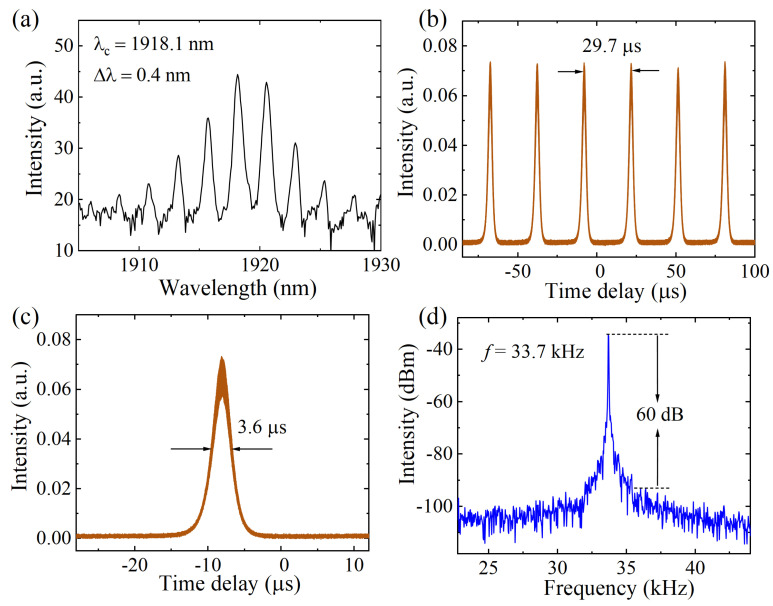
Q-switched results enabled by the Ag/MXene SA in the 2 μm band. (**a**) Spectrum. (**b**) Pulse train. (**c**) Single pulse profile. (**d**) RF spectrum.

Q-switched pulses were generated at the pump power of 160 mW in the 2 μm fiber laser. Figure 4 shows the typical Q-switched results when the pump power was 180 mW, where the output power and pulse energy were 0.86 mW and 25.5 nJ, respectively. As shown in Figure 4a, the Q-switched spectrum had a central wavelength of 1918.1 nm and the full width at half-maximum of the central peak of the spectrum was 0.4 nm. The multiple peaks of the spectrum were due to the nonlinear effect caused by mixing four waves, and the frequencies corresponding to the wavelengths were consistent with the four-wave mixing relationship, which usually occurs under high power in a laser cavity [46,47,48]. Figure 4b depicts the pulse train with a time interval of 29.7 μs, corresponding to the repetition rate of 33.7 kHz. The single pulse profile is shown in Figure 4c, in which the pulse width was 3.6 μs. Figure 4d exhibits the RF spectrum of the Q-switched pulses, and the high signal-to-noise ratio (SNR) of 60 dB indicates the stability of output pulses. Generally, the pulse width of Q-switched pulses are in the order of nanoseconds or microseconds with a high pulse energy. In practical applications, the microsecond pulse width of a Q-switched fiber laser is more attractive for fields such as soft- and hard-tissue ablation and photoacoustic imaging, which need lasers with a long action time and high energy [49]. In addition, the heat conduction and damage of tissue are affected by pulse width in laser medicine surgery, where the pulse width should be selected according to tissue properties to obtain better surgical results [50]. In the surgery process, the fiber sensors are needed to measure the changes in parameters in real time. Moreover, it can be seen from the experimental results that the effect of mixing four waves does not have much impact on the width, energy, or power of the output pulses but mainly affects the shape of the spectrum, where the multi-wavelength output is conducive to its application in fields including frequency conversion and multichannel communication [51,52,53].

Table 1 shows the typical results of the Q-switched fiber laser at 2 μm based on different materials for the SA, including the central wavelength, modulation depth, pulse width, repetition rate, and SNR. The Ag/MXene composite possesses a high modulation depth, which benefits from the enhancement of nonlinear absorption owing to the interaction between Ag and MXene. Generally, an SA with a high modulation depth exhibits a remarkable ability to modulate pulses and suppress noise, thereby narrowing the pulse width and enhancing the pulse stability in lasers. Therefore, the pulse width of the Q-switched pulse based on the Ag/MXene composite was relatively small compared with other results. More importantly, the SNR of the output pulse was up to 60 dB, which is the highest SNR for a Q-switched fiber laser at 2 μm to the best of our knowledge.

**Figure 5:**
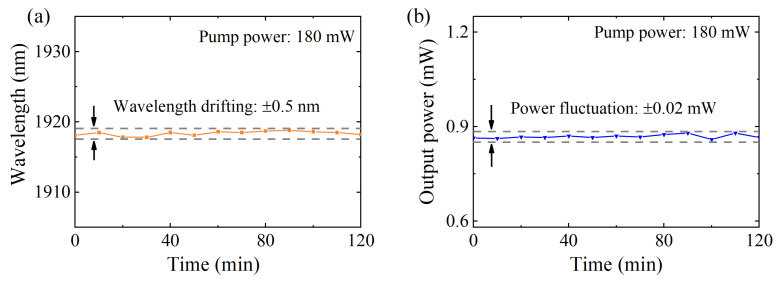
Stability of 2 μm Q-switched fiber laser over 2 h. (**a**) Wavelength and (**b**) output power versus time.

The stability of the Q-switched fiber laser was also monitored over 2 h with a time interval of 10 min. The maximum drifting of the central wavelength in Figure 5a was 0.5 nm. As shown in Figure 5b, the change in output power was also observed, where the maximum fluctuation in output power was only 0.02 mW. It can be seen that the Q-switched fiber laser is robust and reliable in long-term operation.

**Figure 6:**
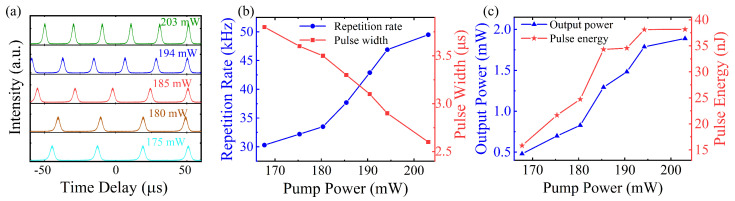
Variation in Q-switched results with the increase in pump power. (**a**) Pulse train. (**b**) Repetition rate and pulse width. (**c**) Output power and pulse energy.

In order to further investigate the properties of Q-switched pulses, the variation in pulse train, pulse width, repetition rate, output power, and pulse energy under different pump powers were measured and analyzed. The pulse trains are shown in Figure 6a with a pump power of 175, 180, 185, 194, and 203 mW. It can be found that the pulse intensity of the Q-switched pulses increased. Additionally, the variations in the detailed parameters of the output pulses were also measured. Figure 6b shows that the pulse width narrowed down and the repetition rate increased with the pump power increase. This trend can be attributed to the improvement in pump efficiency and speed in the gain fiber with the pump power increase, which reduced the time of particle accumulation and enhanced the speed of pulse formation. Consequently, the quantity of pulses generated per unit of time rose, which was equal to the enhanced repetition rate. Simultaneously, more particles were accumulated in the upper energy level, the rate of change in pulse power was accelerated, and the generated pulse became narrower than before. Figure 6c shows the output power and pulse energy under different pump powers. As the pumping efficiency of the gain fiber improved with the increase in pump power, the population of particles accumulated in the upper energy level increased. Consequently, the energy emission per unit time was amplified, leading to a corresponding increase in both the average output power and pulse energy. Therefore, the output power increased to 1.9 mW, and the corresponding single pulse energy was 38 nJ when the pump power reached 204 mW.

Typically, the properties of the material as an SA affect the characteristics of the laser output, and thus composites combining the advantages of different materials have been widely studied in recent years. The enhancement effect of the nonlinear properties of the Ag/MXene composite was explored in this work, where the ability to shape and amplify pulse, as well as noise suppression of the composite, was strengthened based on the combination of Ag and MXene. However, the charge transport between Ag and MXene is a complex transient process, which requires techniques with sufficient temporal resolution or spectral specificity to further observe and explore, such as THz spectroscopy [60,61]. The typical results and the long-term stability of the 2 μm Q-switched laser enabled by the Ag/MXene SA were analyzed, which verified the reliability of the laser. Compared with previous work that investigated the broadband nonlinear responses of Ag/MXene SA in pulsed lasers, this work focused on studying the characteristics of Q-switched pulses generated in a 2 μm Ag/MXene-based laser [62]. Notably, the SNR of the Q-switched pulses reached 60 dB, indicating the higher stability and improved pulse quality under the enhancement effect of the nonlinear properties. In addition, the study of the changes in Q-switched pulse parameters with the pump power was also conducive to control the output characteristics of the laser, further proving its flexibility. The pulse width of the Q-switched pulse narrowed when the repetition rate, output power, and single pulse energy increased with the increase in pump power, further proving the flexibility and controllability of the Q-switched output. Regarding applications, owing to their eye-safe and water absorption waveband characteristics, 2 μm Q-switched fiber lasers have great potential in the fields of medical sensors and laser medicine [63]. Moreover, in laser medicine, the optical fiber sensor should be integrated throughout the entire process to monitor real-time changes in various parameters during the medical process, thereby guiding the surgical operation [49]. The 2 μm Q-switched fiber lasers are reliable light source for fiber sensors in non-destructive testing and non-destructive evaluation, which have the advantages of high precision, high efficiency, non-destructiveness, and comprehensiveness [64,65]. Furthermore, a 2 μm laser is also attractive in tissue imaging as a high-coherence source, such as in optical coherence tomography [66]. The cross section of tissue can be seen in optical coherence tomography, which possesses the advantages of being fast, real-time, dynamic, and non-contact imaging.

## 4. Conclusions

In this work, a Ag/MXene composite was utilized as an SA in a Tm:Ho co-doped fiber laser, where Q-switched pulses were obtained. At the pump power of 180 mW, the output power and pulse energy of stable pulses were 0.86 mW and 25.5 nJ with the repetition rate of 33.7 kHz. The wavelength and output power were monitored over 2 h, where the insignificant fluctuations proved the long-term stability of the laser. In addition, the high SNR of 60 dB indicated the stability of the Q-switched pulses. It can be seen from the comparison that the SNR of the Q-switched pulse was higher than those based on other SA materials, which can be attributed to the enhanced nonlinear properties of the Ag/MXene composite. Furthermore, the properties of output-pulse changes with the pump power were also explored and discussed, where the repetition rate, output power, and single-pulse energy increased, and the pulse width decreased. These results indicate the capacity of the Ag/MXene SA to generate pulses, demonstrating that the Ag/MXene composite is of great significance for broadening its application in ultrafast photonics.

## Figures and Tables

**Table 1:** Typical results of the 2 μm Q-switched fiber laser with different SAs.

Material	Wavelength (nm)	Modulation Depth (%)	Pulse Width (μs)	Repetition Rate (kHz)	SNR (dB)	Reference
Gallium selenide	1986	7.1	6.9	22.9	42	[54]
Holmium oxide	1955	/	2.6	40	47	[55]
Graphene	1976	/	2	31.5	51	[56]
Ag	1928	3.2	4.8	28	33	[57]
Aluminized film	∼1948	3	5.5	37	34	[58]
Pencil-core flakes	1940	12.5	6.7	15	/	[59]
Ag/MXene	1918	14.9	3.6	33.7	60	This work

## Data Availability

Data underlying the results presented in this paper are not publicly available at this time but may be obtained from the authors upon reasonable request.
